# Efficiency and Impact of Hypnoanalgesia for Cardiac Catheterisation in Paediatric Population

**DOI:** 10.3390/jcm12196410

**Published:** 2023-10-09

**Authors:** Pierre-Alexandre Fontanges, Julien Haudiquet, Julien De Jonkheere, Alexandre Delarue, Olivia Domanski, Thameur Rakza, Sebastien Hascoet, Said Bichali, Jean Benoit Baudelet, Francois Godart, Ali Houeijeh

**Affiliations:** 1 Paediatric and Congenital Cardiology Unit, Lille University Hospital, Institut Coeur Poumon, Lille University, UFR3S, Rue Pr. Leclercq, 59000 Lille, France; 2 CIC-IT 1403, Lille University Hospital, 59000 Lille, France; 3 Department of Neonatology, Lille University Jeanne de Flandre Children’s Hospital, Faculty of Medicine, University of Lille, F-59000 Lille, France; 4 Department of Pediatric Cardiology, Marie Lannelongue Hospital, 92350 Le Plessis-Robinson, France; 5 Evaluation of Health Technologies and Medical Practices (METRICS)-ULR 2694, University of Lille, F-59000 Lille, France

**Keywords:** MeSH terms: hypnoanalgesia, hypnosis, congenital heart disease, cardiac catheterisation, paediatric population

## Abstract

Hypnoanalgesia is a promising non-pharmacologic adjunct technique in paediatric interventions. Its safety, efficiency, and impacts on paediatric cardiac catheterisation (CC) are unknown. Methods: In a prospective study, patients aged <16 years who underwent CC under hypnoanalgesia from January to December 2021 were included. Pain and anxiety were assessed using the analgesia nociception index (ANI) and the visual analogue scale (VAS). Results: Sixteen patients were included; the mean age was 10.5 years, and the mean weight was 37 kg. Catheterisations were interventional in 10 patients (62.5%). Hypnoanalgesia indications were general anaesthesia (GA) contraindication in four patients (25.0%), the need for accurate pressure measurements in three patients (18.7%), and interventionist/patient preferences in nine (56.3%). CC was accomplished in 15 patients (93.7%), even in complicated cases. In one case, pulmonary artery pressures were normalised compared to previous catheterisation under local anaesthesia alone. The VAS score was under 5/10 for all patients. The ANI remained above 50 (no painful zone) for all but one patient. There was no significant decrease in the ANI during the intervention compared to the baseline (*p* = 0.62). No complications were reported. Conclusion: Paediatric CC is feasible and safe under hypnoanalgesia, even in complicated cases. Hypnoanalgesia was efficient in managing pain and stress, and it ensures more reliable pressure measurements.

## 1. Introduction

In patients with congenital heart disease, interventional indications increase steadily. At the same time, diagnostic catheterisations remain mandatory in some cases, such as pulmonary vascular resistances and vascular reactivity assessment. Nevertheless, cardiac catheterisation (CC) has some limitations in children because it is an anxious and painful procedure, which requires general anaesthesia (GA) in most cases. Previous studies have shown adverse neurocognitive effects and a decrease in school performance after repeated administration of GA [1,2]. Moreover, GA drugs and interventions, such as endotracheal intubation, can be very challenging, mainly in children with severe congenital heart disease and heart failure, and in some patients, they may be contraindicated due to high medical risk [3]. Third, GA and ventilation modify cardiopulmonary interaction and intrathoracic pressure and require oxygen supplementation. Altogether, these factors induce significant haemodynamic modifications and thereby induce major errors in measurements [4]. Finally, GA requires more human and material resources, and it is consequently more expensive. 

Another challenging issue in paediatric CC is pain control. It is known that pain can induce long-term harmful effects on neurologic, psychological, and haemodynamic conditions [5]. Additionally, the quality of life of a patient and attachment to medical care could be altered by a negative and painful experience. However, pain is sometimes difficult to evaluate objectively in children. Some methods, such as the visual analogue scale (VAS) [6] or analgesia nociception index (ANI) (MDoloris Medical Systems, Loos, France) [7], have been developed to measure discomfort felt by patients. The ANI already showed its ability for nociception/antinociception imbalance evaluation both in conscious patients and those under GA [8,9].

Hypnoanalgesia is one of the various mind–body therapies that influence the perception of oneself and modify the state of consciousness [9]. Usually, the hypnotherapist guides the interaction with the patient to encourage them to fix their attention to an interior experience. These mind–body therapies are already used in children with bowel syndrome and nocturnal enuresis and for the treatment of chronic or functional pain [10]. Several publications have demonstrated the efficiency of clinical hypnoanalgesia for invasive procedures in adult cardiac interventions, such as angioplasty or cardiac arrhythmia transcatheter ablation [11]. To the best of our knowledge, only one experience of clinical hypnoanalgesia for cardiac procedures has been published. Amedro et al. described their experience of 16 transoesophageal echocardiography procedures in children and adolescents with very promising results [12].

Hypnoanalgesia and pain reduction could increase children’s compliance and procedure success rates and improve the quality of life of paediatric patients. 

However, the feasibility, safety, and effectiveness of hypnoanalgesia are not proven for paediatric CC. The aim of this study was to ensure that hypnoanalgesia is safe and effective for CC in children. 

## 2. Objective

Our objective was to evaluate the feasibility, safety, and impact of clinical hypnoanalgesia associated with local anaesthesia in paediatric catheterisation procedures. 

## 3. Methods

### 3.1. Study Design and Population 

This prospective observational practice evaluation study (CAPHYPNO; CAtheterisation cardiaque Pédiatrique sous HYNPO-analgésie) was carried out over 1 year (from January to December 2021) in a tertiary care paediatric and congenital cardiology referral centre (Lille University Hospital, Lille, France). National Commission for Information Technology and Civil Liberties (CNIL) authorisation was obtained (DEC21-348).

All children aged 4 to 18 years requiring diagnostic or interventional CC were eligible for this study. Patients under 4 years were not included because of the absence of adequate verbal communication and symbolisation needed for hypnoanalgesia. 

Hypnoanalgesia was proposed only for indications already practiced under local anaesthesia in adults at our centre.

Afterwards, the children and their parents or legal guardians were given detailed information about the CC and clinical hypnoanalgesia by qualified staff. Then, patients were examined by a hypnotherapist and anaesthesiologist to test their sensory channels, receptivity, and preferred thematic to validate the possibility of the hypnoanalgesia, and to anticipate the necessity of more sedation in case of procedure failure due to pain or anxiety. The anxiety level before the intervention was determined by the hypnotherapist as low, moderate, or high. 

### 3.2. Hospitalisation Management

Children were hospitalised the day before the procedure. Peripheral venous access was obtained under hypnoanalgesia by the same caregiver that performed hypnoanalgesia during catheterisation. All procedures were performed by the same hypnotherapist. The hypnotherapist was a nurse with a nationally validated certification. The hypnoanalgesia certification program is a 1-year program composed of courses and practical formation hours of training by companionship. Formation training was financed by our unit. 

Before the intervention, medical teams detailed the most painful moments of the intervention, which require the deepest analgesia. 

On the day of the procedure, the hypnotherapist escorted the patient from his/her hospital room to the operative room to reassure them and decrease the separation stress. An ANI monitor was plugged into the cathlab haemodynamic monitoring system to evaluate pain/analgesia using a measurement algorithm based on the magnitude analysis of the respiratory patterns and heart rate variability. ANI registration started before the patient was introduced to the cathlab to determine the baseline ANI. Hypnoanalgesia was initiated. All interventions were performed by the same interventionist (AH). After a positive sign from the hypnotherapist, local anaesthesia with xylocaine (10 mg/mL; 2 to 5 mg/kg) was administered, and nitrous oxide inhalation was introduced until the end of the puncture as usually performed for procedures under local anaesthesia alone. The hypnotherapist guided the interaction with the patient’s preferred thematic to encourage them to fix their attention to an interior experience, to disconnect from the reality of the cathlab. Interaction was also maintained between the interventionists and the hypnotherapist to anticipate possible painful moments during the intervention that require the deepest hypnoanalgesia. Therefore, the intervention was conducted as usual at our centre. The atrial septal defect (ASD) was measured using an Amplatzer sizing balloon (Abbott, Chicago, IL, USA) or Meditech Equalizer balloon catheter (Boston, MA, USA). A double-disc nitinol device was used to close the ASD under transthoracic echocardiography as is frequently performed in adults at our centre. Persistent ductus arteriosus (PDA) was assessed using a lateral view angiogram. PDA was closed with a coil (Cook, Bloomington, IN, USA) for small ductus <2 mm or nitinol-based devices for larger ones. As much as possible, the registered fluoroscopies with contrast injection were preferred over angiographies to decrease the irradiation dose and allow more radioprotection for the patient and hypnotherapist. Rather than the lead apron, additional radioprotection tools (radiation cap, lead glasses, and plumbed shield) were provided to the hypnotherapist because of his/her position beside the head of the patient and proximity to the X-ray generator.

### 3.3. Study Outcomes 

The quantitative data from the interventions were collected to assess the success rate and impact of hypnoanalgesia on procedure times. The need for more sedation and complications were noted. These data were compared with similar interventions performed by the same primary interventionist during the same period. To assess the effect of hypnoanalgesia on procedure times, a control group was selected and matched to the hypnosis group using a propensity score based on patient age and intervention type. Otherwise, the idea of randomisation to create a paediatric control group under local anaesthesia alone was not accepted by the ethical commission of our institution. 

Pain assessment was performed using the ANI monitor. The ANI was recorded along the duration of the procedure until the end of the vascular compression. Objective pain assessment was performed by evaluating the time spent with an ANI value lower than 50, which corresponds to the threshold defined by the manufacturer for detecting a significant pain response. ANI values 2 min after the xylocaine administration and 2 min after the vascular puncture (the most painful procedure) were registered to analyse the analgesic effect of hypnoanalgesia. Further analyses were performed on the highest score of the ANI. The ANI represents the score after the painful steps were performed. Otherwise, after the procedure, patients gave their own pain evaluation using the VAS [13].

A qualitative evaluation of the procedure was performed using several methods. A satisfaction survey was filled out by the patient and/or his/her family after the procedure regarding their level of satisfaction with the use of catheterisation under hypnoanalgesia. 

### 3.4. Statistics 

Data analysis was descriptive. Numerical data were expressed as median (first to third quartile). Differences in ANI values after xylocaine administration and after vascular puncture were assessed using a Wilcoxon non-parametric test. *p* > 0.05 was considered statistically significant. 

For the qualitative evaluation, a survey with several questions was used to ask about the information given to the patient regarding hypnoanalgesia, their feelings about undergoing such intervention under hypnoanalgesia, and whether they would recommend this technique to other patients.

## 4. Results 

### 4.1. Patient Data (Table 1)

Among the 16 patients included, 6 were boys and 10 were girls. The median age was 11 years (range 4–16); catheterisation was interventional in 10 patients (62.5%) and for diagnostic purposes in 6 patients (37.5%). Interventional catheterisation indications were ASD closure in six patients, PDA closure in three patients, and electrophysiological ablation in one patient. Diagnostic catheterisation indications were precardiac transplantation evaluation in one patient with severe dilated cardiomyopathy, pulmonary hypertension evaluation in one patient, pulmonary vein stenosis suspicion in one patient who had been operated on for anomalous pulmonary venous return with recurrent clastic pneumonia, one patient with coronarography, one patient with borderline aortic coarctation, and one patient with Fontan failure.

Hypnoanalgesia indications were challenging or GA was contraindicated in four patients: one 14-year-old patient with Fontan failure with heterotaxia, an unbalanced atrioventricular septal defect, severe right ventricle hypoplasia, severe left ventricle dysfunction, exudative enteropathy, deep cyanosis, and psychological difficulties; one 11-year-old patient with dilated cardiomyopathy and severe left ventricular dysfunction; recurrent clastic pneumonia in one patient; and a 12-year-old girl with metabolic disorders (homocystinuria).

In two patients, hypnoanalgesia was deemed necessary to obtain reliable pressure measurements to avoid GA interference. One patient had borderline coarctation and mild transverse arch hypoplasia, and the other patient had mitral valve disease and pulmonary hypertension diagnosed during a previous catheterisation under local anaesthesia alone. For 10 patients, hypnoanalgesia indication was preferred by the family/surgeon. No contraindication for the catheterisation under hypnoanalgesia was issued by the anaesthesiologists after their evaluation. 

Eleven patients (68.7% of the patients) had light or moderate anxiety before the procedure, and only three patients showed severe anxiety. The stress level was not assessed in the other patients due to the absence of a response to the satisfaction survey.

**Table 1:** Patients’ characteristics.

Patients	Age (years)	Sex	Weight (kg)	Procedure Indication	Hypnosis Indication	Anxiety before Procedure	Clinical Hypnosis Success	Patient/ Family Satisfaction	ANI Score (mean)
1	16	F	48	ASD closure	Family choice	Low	Yes	Neutral	49
2	8	M	21	Diagnostic catheterisation/pulmonary hypertension	Reliable haemodynamic assessment	Moderate	Yes	Yes	
3	7	F	18	ASD closure	Family choice	Important	Yes	Yes	86
4	11	M	48	Diagnostic catheterisation/pulmonary hypertension	GACI	-	Yes	Yes	75
5	16	F	74	ASD closure	Family choice	Important	Yes	Yes	52
6	4	M	20	PDA closure	Family choice	Low	Yes	Yes	70
7	16	F	50	Coronarography	GACI	Moderate	Yes	Yes	69
8	7	F	21	ASD closure	Family choice	Low	Yes	Yes	92
9	12	F	43	Diagnostic catheterisation/aortic coarctation	Reliable haemodynamic assessment	Low	Yes	Yes	67
10	16	M	79	Electrostimulation procedure	Family choice	Low	Yes	Yes	
11	4	M	15	PDA closure	Family choice	Moderate	No	No answer	
12	8	F	46	Diagnostic catheterisation/anomalous pulmonary venous return/aortic coarctation	Reliable haemodynamic assessment/GACI	Low	Yes	Yes	79
13	14	F	25	Diagnostic catheterisation/Fontan failure	Reliable haemodynamic assessment/GACI	-	Yes	Yes	88
14	12	F	47	PDA closure	Family choice	-	Yes	No answer	64
15	6	M	19	ASD closure	Family choice	Low	Yes	Yes	81
16	11	M	30	ASD closure	Family choice	Low	Yes	Yes	97

ASD: atrial septal defect, PDA: persistent ductus arteriosus, GACI: general anaesthesia contraindication.

### 4.2. Feasibility and Security

Hypnotic status was obtained in 15 out of the 16 patients. In a 4-year-old patient, hypnotic status was not reached despite the favourable evaluation during the previous hypnoanalgesia consultation. This result was expected because of the failure of the peripheral vein puncture under hypnoanalgesia the day before the catheterisation. Finally, the procedure was aborted before the installation in the cathlab.

Otherwise, hypnoanalgesia and catheterisations were performed successfully in the other patients (success rate, 93.7%). Several patients were challenging. In the previously described 14-year-old girl with Fontan failure and psychological difficulties, a long (75 min) complete left and right heart catheterisation was performed with angiograms to search for collaterals and real-time oxygen consumption measurement to evaluate the resistance of pulmonary arteries and cardiac index. A large ASD was closed with a 39 mm Occlutech Figulla Flex (Occlutech, Sweden) device using the left pulmonary vein technique in a 16-year-old patient. A 7-year-old patient with a large ASD (26 mm/m^2^) had experienced a failed surgery attempt 2 years prior due to the large size of the ASD, which was performed under GA. The ASD was closed successfully with an 18 mm Amplatzer Septal Occluder (Abbott, USA). 

Median procedure (puncture to compression) and fluoroscopy times were 33.5 min (interquartile range 20.5–43.7) and 4.0 min (interquartile range 3.0–6.0), respectively. These procedure and irradiation times were comparable to those of similar patients who had interventions under general anaesthesia (*p* = 0.85 and *p* = 0.91 respectively).

No complication was reported in any patient. 

### 4.3. Efficiency 

No additional medication or sedation was necessary in any of the patients. In the patient with mitral valve disease and pulmonary hypertension diagnosed during a previous catheterisation (mean pulmonary artery pressure (mPAP) 31 mmHg), the decision was made to control the pulmonary pressure 1 year later because of the contradiction between his clinical status and haemodynamic evaluation. Pulmonary pressures were normal under hypnoanalgesia (mPAP 20 mmHg), leading to the cancellation of the cardiac surgery of the mitral valve. 

The median hospitalisation cost was reduced by EUR 455, from EUR 3880 to EUR 3425 (12.8%).

### 4.4. Effectiveness of the Hypnoanalgesia on Pain Control

The ANI signal had good quality in 13 patients. The amount of time spent with an ANI lower than 50 was 0.05% (0.3–19.0). 

**Figure 1:**
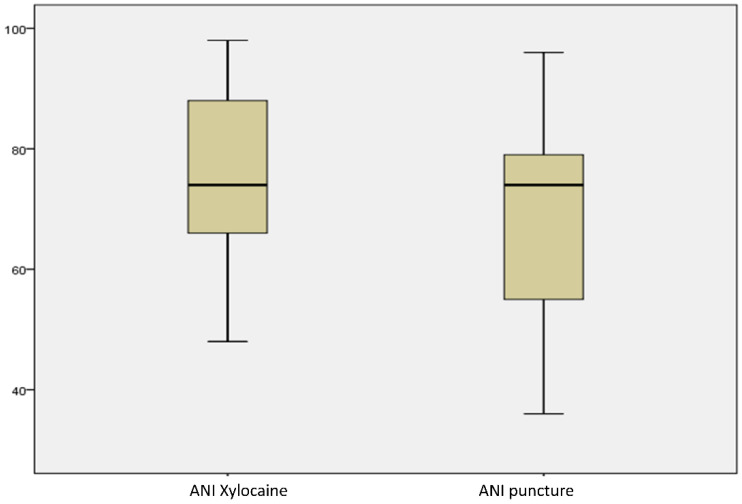
Anaesthesia nociception index (ANI) before and after the vascular puncture. Results are expressed as mean +/− standard deviation. *p* = 0.44.

The ANI remained above 50 after the vascular puncture with a median value of 75 (IQT 67–86) in all patients except one who presented with an ANI value of 49 after the procedure. This 16-year-old patient with ASD had language difficulties with additional stress because of nudity. The ANI dropped from 70 to 49 for a short time (1 min) after the puncture with quick normalisation. The modification of the ANI score between the baseline at the vascular puncture time, considered as the most painful event, was not significant (79 [IQT 67; 86] vs. 75 [IQT 65.5; 87], respectively, *p* = 0.441) (Figure 1). Pain assessment with the VAS was performed for 12 patients, and the score was under 3 (no painful zone) for 8 patients (66.6%) and between 4 and 6 for 4 patients (33.3%). No patient had a score above 6, which corresponds to highly painful. 

Satisfaction: Out of the 16 patients, 14 (87.5%) were satisfied or very satisfied, 1 did not respond, and 1 was neutral on his answer (Table 2).

## 5. Discussion 

CC is a mandatory investigation in some children. CC in children needs complex management to obtain reliable results and to avoid the harmful consequences of pain in this population. Hypnoanalgesia is an interesting tool for managing pain and anxiety during invasive interventions. It is often practiced based on caregivers’ sporadic experiences. To the best of our knowledge, this is the first study that objectively reports the safety, feasibility, and advantages of clinical hypnoanalgesia in CC in the paediatric population. In this monocentric study at a tertiary centre for 1 year, CCs were performed with feasible hypnoanalgesia and local anaesthesia in most cases, even in complex ones without complications. The objective assessment of pain using the ANI demonstrated that hypnoanalgesia was effective in controlling pain and anxiety during the intervention. Moreover, hypnoanalgesia allowed a more accurate haemodynamic assessment. Finally, this practice improved family/patient satisfaction and reduced hospitalisation costs. 

### 5.1. Feasibility and Safety of CC under Hypnoanalgesia 

In the last few decades, indications for interventional CC have been increasing in children and adolescents with congenital cardiac diseases, owing to the improvement in techniques and the development of smaller devices [14]. GA is usually recommended in this population because of the lack of patient compliance and the need for optimal sedation and pain control. However, GA has several limitations, such as pressure modifications, medical haemodynamic and neurologic risks, and costs [4]. Additionally, several stressful moments are not covered by GA, like separation from the family, the steps before the beginning of GA, and the period in the recovery room. In this study, most CCs were feasible under hypnoanalgesia, even in challenging cases without additional medical sedation or modification of the procedure duration and complications. Moreover, hypnoanalgesia was very beneficial in four patients for whom GA was considered highly risky. These encouraging results correspond to the results from the recently published study on hypnoanalgesia in transoesophageal echocardiography by Amedro et al. [12], proving that hypnoanalgesia is particularly useful in patient care in our paediatric population. Similarly, several complex transcatheter cardiac procedures, such as valve replacements, were performed under hypnoanalgesia in adults [15]. 

To the best of our knowledge, this was the first study including children. Several factors contributed to this success: physicians preparing the family, the presence of qualified caregivers who had specific training, and the combined consultation of the hypnotherapist with the anaesthesiologist. The routine practice of hypnoanalgesia for minimally invasive interventions, such as peripheral vein puncture, surgical drains withdrawal, and bandage care, is performed in our unit. The teamwork reassures the interventionists and the patients and their families. In contrast, it is noteworthy to emphasise that interventional catheterisations under hypnoanalgesia are routinely performed under local anaesthesia in adults at our centre. Consequently, the success rate was not impacted by the replacement of GA with hypnoanalgesia in children. In the same way, the interventional and fluoroscopy durations were similar to those under GA. 

### 5.2. Efficiency of the Hypnoanalgesia in the Haemodynamic Assessment 

Haemodynamic assessment remains one of the main indications for catheterisation. However, in most cases, GA is used in children, which mostly requires drug usage, ventilation, and oxygen supply. All these factors modify the haemodynamic conditions. Similarly, stress and pain in the case of local anaesthesia modify the haemodynamic conditions. Therefore, finding the most efficient method to measure haemodynamic pressure at rest as recommended is a real dilemma for paediatric surgeons [4]. Hypnoanalgesia seems to be a very efficient tool. The ANI registration during the interventions confirmed the absence of pain and stress as interfering factors. In one patient in this study, the pulmonary pressures were elevated in a previous catheterisation under local anaesthesia alone, and they were measured as normal during the control under hypnoanalgesia, with a real impact on decision making. Consequently, hypnoanalgesia seems to be a safe and interesting alternative to GA for haemodynamic assessment in children aged more than 4 years.

### 5.3. Efficiency of the Hypnoanalgesia in Pain and Stress Management 

Patient compliance and quality of life could be altered by painful interventions, therapeutic strategies, and hospitalisation stress [16,17]. In addition, pain may alter neurologic and psychological development [2]. GA could also impact the neurologic outcome in children [10]. Finally, with the improvement in our patient survival rate, special attention is nowadays paid to quality of life and neurologic outcomes [18]. Therefore, pain control is one of the milestones of paediatric patient management [17]. Moreover, several psychological factors are known to modify pain experience, such as anxiety and fear of the unknown, leading to hypervigilance by the patient [19]. CC includes several stressful moments, such as the announcement, hospitalisation, separation from the family to go to the cathlab, preparation of the procedure, and certainly the procedure itself in case of local anaesthesia. Altogether, these moments could induce significant stress, which could impact the patient’s perception and quality of life. However, in the case of GA, all stressful moments around the intervention could be unconsciously neglected. The hypnoanalgesic protocol practiced for this population allowed the global management of stress and painful events. Patient management began at the preinterventional visit; thereafter, the hospitalisation and separation stress was also covered by the hypnoanalgesia offered by the same caregiver. This global management leads to breaking the pain encoding schema and the vicious cycle of pain [19]. In our study, the hypnoanalgesia protocol was efficient in reducing patient pain and stress. During the intervention, the ANI and VAS scores demonstrated the absence of pain or anxiety in most patients. Consequently, the satisfaction survey after the intervention demonstrated an excellent level of satisfaction among the patients and their families. Relying on the substrate of emotional support and empathy with the patient, hypnoanalgesia may therefore improve the patient’s quality of life, as it has been proven for other chronic diseases [10]. Hypnoanalgesia constitutes an effective alternative to avoid pain and stress from the announcement of the intervention and during the whole hospitalisation time.

Hypnoanalgesia provides other benefits in addition to the clinical ones: the calm and serenity in the cathlab and the impact on the irradiation dose. In fact, the presence of the hypnotherapist beside the patient makes the surgeons reduce the irradiation dose, preferring saved fluoroscopies instead of angiograms. Furthermore, hypnoanalgesia is less expensive than GA, leading to a cost reduction of approximately 13% [11]. More importantly, there is less need for human resources (anaesthesiologists and anaesthesia nurses) as well as medical materials. This advantage is especially relevant because of the lack of medical human resources in France and Europe. 

### 5.4. Hypnoanalgesia Limits 

Hypnoanalgesia has several limitations in the paediatric population. It cannot be performed in patients under 4 years of age due to the barrier in communication. Also, a multidisciplinary consultation, associating a hypnotherapist and anaesthesiologist, is needed. The hypnotherapist needs to have experience and training in the specified area, and at the moment, only a limited number of physicians are trained in this field. Finally, the radioprotection of the hypnotherapist could be challenging because the hypnotherapist needs to be in constant contact with the patient. The development of techniques, such as virtual reality, for the hypnotherapist–patient contact could be a future solution. 

### 5.5. Study Limitation 

This was a single-centre non-randomised study with a small sample size. All the interventions were performed by the same surgeon and hypnotherapist; therefore, no data were available to show large-scale feasibility. There was no control group, stress level was not assessed in other patients without hypnoanalgesia.

## 6. Conclusions 

Paediatric CC is feasible under hypnoanalgesia associated with local anaesthesia, even in technically complicated cases or in cases where GA is contraindicated. Hypnoanalgesia ensures more reliable pressure measurements and is efficient in managing pain and stress before and during the procedure in most cases, improving the satisfaction of patients and their families.

## Figures and Tables

**Table 2:** Patient/family satisfaction.

Patient/Family Satisfaction	Number
Very satisfied or satisfied	13
Neutral	1
Not satisfied	0
No answer	2
Total	16

## Data Availability

All the authors certify that the most relevant data are included in the article. Moreover, the authors agree to provide all requested supplementary data (e.g., the ANI data or other pain assessment tools) after a simple message to the corresponding author. Obviously, patient privacy and the ethical rules of the medical institutions will be respected in the treatment of the data.

## Abbreviations

CC	cardiac catheterisation
GA	general anaesthesia
VAS	visual analogue scale
ANI	analgesia nociception index
ASD	atrial septal defect
PDA	persistent ductus arteriosus
